# *In Situ* complement activation and T-cell immunity in leprosy spectrum: An immunohistological study on leprosy lesional skin

**DOI:** 10.1371/journal.pone.0177815

**Published:** 2017-05-15

**Authors:** Nawal Bahia El Idrissi, Anand M. Iyer, Valeria Ramaglia, Patricia S. Rosa, Cleverson T. Soares, Frank Baas, Pranab K. Das

**Affiliations:** 1 Department of Genome Analysis, Academic Medical Center, Amsterdam, The Netherlands; 2 Department of Neuropathology, Academic Medical Center, Amsterdam, The Netherlands; 3 Instituto Lauro de Souza Lima, Bauru, Brazil; 4 Department of Clinical genetics, Leiden University Medical Center, Leiden, The Netherlands; 5 Department of Clinical Immunology, Colleges of Medical and Dental Sciences, University of Birmingham, Birmingham, United Kingdom; University of Minnesota, UNITED STATES

## Abstract

*Mycobacterium leprae* (*M*. *leprae*) infection causes nerve damage and the condition worsens often during and long after treatment. Clearance of bacterial antigens including lipoarabinomannan (LAM) during and after treatment in leprosy patients is slow. We previously demonstrated that *M*. *leprae* LAM damages peripheral nerves by *in situ* generation of the membrane attack complex (MAC). Investigating the role of complement activation in skin lesions of leprosy patients might provide insight into the dynamics of *in situ* immune reactivity and the destructive pathology of *M*. *leprae*. In this study, we analyzed in skin lesions of leprosy patients, whether *M*. *leprae* antigen LAM deposition correlates with the deposition of complement activation products MAC and C3d on nerves and cells in the surrounding tissue. Skin biopsies of paucibacillary (n = 7), multibacillary leprosy patients (n = 7), and patients with erythema nodosum leprosum (ENL) (n = 6) or reversal reaction (RR) (n = 4) and controls (n = 5) were analyzed. The percentage of C3d, MAC and LAM deposition was significantly higher in the skin biopsies of multibacillary compared to paucibacillary patients (p = <0.05, p = <0.001 and p = <0.001 respectively), with a significant association between LAM and C3d or MAC in the skin biopsies of leprosy patients (r = 0.9578, p< 0.0001 and r = 0.8585, p<0.0001 respectively). In skin lesions of multibacillary patients, MAC deposition was found on axons and co-localizing with LAM. In skin lesions of paucibacillary patients, we found C3d positive T-cells in and surrounding granulomas, but hardly any MAC deposition. In addition, MAC immunoreactivity was increased in both ENL and RR skin lesions compared to non-reactional leprosy patients (p = <0.01 and p = <0.01 respectively). The present findings demonstrate that complement is deposited in skin lesions of leprosy patients, suggesting that inflammation driven by complement activation might contribute to nerve damage in the lesions of these patients. This should be regarded as an important factor in *M*. *leprae* nerve damage pathology.

## Introduction

Leprosy is a chronic granulomatous disease caused by the intracellular bacterium *Mycobacterium leprae (M*. *leprae)* which displays a broad spectrum of immunological and histopathological responses. The leprosy spectrum has as its poles either tuberculoid (TT) or lepromatous (LL), and intermediate forms known as borderline lepromatous (BL), borderline borderline (BB) and borderline tuberculoid (BT). The LL, BL and BB forms are collectively called multibacillary (MB) whereas the BT and TT are paucibacillary (PB) [1]. Histopathologically, TT skin lesions are characterized by the presence of epithelioid cells surrounded by a cuff of T-cells with few or no bacilli, whereas LL lesions show an abundance of bacilli-filled foamy macrophages.

The immunopathological spectrum in leprosy is largely considered to be due to the variation in immune responses accompanied with changing granulomatous reactions by the individual host to specific *M*.*leprae* antigens. Tuberculoid leprosy is characterized by a strong T-cell-mediated immunity towards the antigens of *M*. *leprae* whereas lepromatous leprosy is characterized by a selective T-cell unresponsiveness to *M*. *leprae* antigens [2]. In contrast, high levels of *M*. *leprae* specific antibodies are present in LL which does not prevent the spread of the bacteria within the host. The borderline forms of leprosy (BT, BB and BL) are immunologically unstable. In addition, about 20–30% of the borderline patients may undergo immune exacerbations during the course of the disease, which manifest as either reversal reaction (RR) or erythema nodosum leprosum (ENL). This event can follow initial treatment and could worsen the nerve damage even after release from treatment.

Most studies have shown that the involvement of the adaptive immunity is responsible for tissue destruction in leprosy. However, recent evidence suggests that the innate immunity of the host including complement activation plays an important role in leprosy pathology and tissue destruction.

The complement system is the first line of defence against pathogens and a key component of innate immunity, activated early after infections. Activation of the complement system can occur via the recognition of antigen-antibody complexes (classical pathway), foreign surfaces (alternative pathway) or bacterial sugars (lectin pathway). Regardless of the trigger, activation results in the cleavage of C3, and formation of the membrane attack complex (MAC), which lyses cells by making holes in their membrane. Activated complement is able to drift from the target site to adjacent areas and enhance inflammation and damage healthy tissue [3, 4].

The complement system is crucial for the opsonisation and subsequent killing of bacteria. Previous studies have indicated an important role for complement in leprosy, showing increased levels of complement components by serological and pathological studies [5–11]. Another study showed deposits of the MAC in cutaneous sensory nerves of leprosy patients, suggesting a possible role for MAC in leprosy pathology [10, 12]. We have shown that formation of the MAC contributes to early demyelination and axonal damage after traumatic injury of the peripheral nerve [13, 14], and that inhibition of MAC formation reduces nerve damage [15] and improves regeneration and functional recovery [16].

The pathogenesis of nerve damage in leprosy patients remains largely unsolved. We have shown that complement contributes to peripheral nerve damage in a model of *M*. *leprae* induced neuropathy [17]. The interesting question is what triggers the extensive nerve damage. Important elements of an infection with *M*. *leprae* are the recognition of pathogen associated molecular patterns, such as LAM, by pattern recognition receptors that can trigger the activation of the complement system. In nerve biopsies of leprosy patients we found a correlation between the amount of MAC and *M*. *leprae* antigen LAM deposition, suggestig that LAM is a trigger for complement activation [17]. An other study showed an increased amount of antibodies against bacterial antigens such as Lipoarabinomannan (LAM) in serum of multibacillary patients compared to paucibacillary patients [18, 19], suggesting an immune response to the bacterial antigens.

Persistence of *M*. *leprae* antigens from dead bacilli can provoke immunological reactions, such as reversal reaction, causing serious nerve damage and subsequent disabilities. Although multiple drug therapy (MDT) is an affective target to kill *M*. *leprae*, early diagnosis and an effective treatment of the disease related nerve damage is still a challenge. Treatment with MDT targets *M*. *leprae* and this consequently results in reduction of viable bacilli, and initiates the release of dead bacilli and *M*. *leprae* antigens. This could cause a persistent stimulus with consequent activation of the complement system and continued inflammatory response, which contributes to nerve damage. Others and we showed that bacterial antigens such as LAM and axonal debris could be found in leprosy patients long after treatment [20]. Persistence of *M*. *leprae* antigens might be an important risk factor for late reactions by continuously triggering pathogen recognition receptors and activation of complement.

It is important to understand what role the complement system has in leprosy, because increasing evidence suggests that complement is not only involved in killing of pathogens but also plays a critical role in modulating the adaptive immune response and causing nerve damage. Understanding the role of the complement system in the immunopathology in leprosy skin lesions could be of benefit to develop therapeutic intervention in modulating the course of the disease.

This study gives an insight into the immunopathology in skin lesions of leprosy patients throughout the spectrum. It is unknown whether the presence of the *M*. *leprae* antigen LAM is associated with the amount of complement activation in skin lesions of leprosy patients. Here we explore to what extent complement is present in skin lesions of paucibacillary, multibacillary patients and patients with a reaction (ENL and RR) in relation to the presence of *M*. *leprae* antigen LAM. We analyzed borderline lepromatous leprosy patients that developed ENL or RR. In addition, we were interested in the cellular localization of complement activation products C3d and MAC and whether MAC targets the axons in skin lesions of leprosy patients.

Our data supports the hypothesis that the persistence of LAM in leprosy lesions could be the driver of perpetuating disease fluctuation. We propose that complement plays a significant role in inflammation not only through the deposition of tissue damaging complement activation product MAC but also via the involvement of the infiltrating T-cells *in situ*.

## Materials and methods

### Skin biopsies

Skin biopsies of paucibacillary (TT and BT) (n = 7) and multibacillary (BL and LL) (n = 7) leprosy patients were from Brazilian donors and were obtained at hospitalization at the Instituto Lauro de Souza Lima, Bauru, Sao Paulo, Brazil as diagnostic procedure (**Table 1**). In this study we did not include any borderline boderline (BB) patient as this group is unstable and rare, which makes pathological diagnosis difficult. Skin biopsies of leprosy patients after treatment (BL) (n = 4) or with erythema nodusum leprosum (ENL) (n = 4) and reversal reaction (RR) (n = 4) were obtained from the archieval material of the Academical medical Center and were from Dutch donors (**Tables 2 and 3**). The reaction patients were BL patients that developed ENL or RR. We choose BL leprosy patients because they can develop ENL as well as RR. All patients were classified according to the Ridley-Jopling scale. The control biopsies (n = 4) were obtained during surgery from patients with no leprosy. All material was previously obtained for routine diagnostic procedures and made available for this study by the diagnostic pathology laboratory of the AMC. Only material leftover after the diagnostic procedures were completed was used. All samples were de-identified. This study has been approved by the Medical Ethical Committee (METC) of the Academic Medical Center, Amsterdam, the Netherlands for the use of archival material according to Dutch law (WBMO, article 476). Written informed consent was obtained from all the patients.

**Table 1:** Characterization of skin biopsies and clinical data of PB/ MB leprosy patients and controls.

Case	Material	Leprosy type	Gender	Age diagnosis	Treatment
1	Skin	-	F	43	-
2	Skin	-	M	52	-
3	Skin	-	F	26	-
4	Skin	-	M	31	-
5	Skin	-	M	38	-
6	Skin	Paucibacillary (TT)	F	12	MDT/PB (2009)
7	Skin	Paucibacillary (TT)	M	unkown	MDT/PB (2009)
8	Skin	Paucibacillary (BT)	F	29	MDT/ (2010)
9	Skin	Paucibacillary (BT)	F	49	MDT/MB (2010)
10	Skin	Paucibacillary (BT)	F	53	MDT/PB (2010/11)
11	Skin	Paucibacillary (TT)	M	23	unknown
12	Skin	Paucibacillary (BT)	M	29	unknown
13	Skin	Multibacillary (LL)	M	unknown	MDT/MB (2010/11)
14	Skin	Multibacillary (LL)	F	28	MDT/MB (2009/10)
15	Skin	Multibacillary (BL)	F	49	MDT/MB (2009/10)
16	Skin	Multibacillary (LL)	M	39	MDT/MB (2010/11)
17	Skin	Multibacillary (LL)	M	28	MDT/MB (2010)
18	Skin	Multibacillary (LL)	M	79	MDT/MB (2010/11)
19	Skin	Multibacillary (BL)	F	26	MDT/MB (2010)
20	Skin	Multibacillary (BL)	M	46	MDT/MB (2010)
21	Skin	Multibacillary (BL)	F	71	MDT/MB (2010)
22	Skin	Multibacillary (BL)	M	50	MDT/MB (2010)

F, female; M, male; MDT, multidrug therapy

**Table 2:** Characterization of skin biopsies and clinical data of RR leprosy patients.

Case	Material	Leprosy type	Gender	Age diagnosis	Treatment
1	Skin	Multibacillary (RR)	M	36	MDT/MB (1998)
2	Skin	Multibacillary (RR)	F	57	MDT/MB (1995)
3	Skin	Multibacillary (RR)	M	40	MDT/MB (1996)
4	Skin	Multibacillary (RR)	F	40	MDT/MB (1997)

F, female; M, male; MDT, multidrug therapy

**Table 3:** Characterization of skin biopsies and clinical data of ENL leprosy patients

Case	Material	Leprosy type	Gender	Age diagnosis	Treatment
1	Skin	Multibacillary (ENL)	F	28	MDT/MB (2009–10)
2	Skin	Multibacillary (ENL)	M	39	MDT/MB (2010–11)
3	Skin	Multibacillary (ENL)	F	18	MDT/MB +PRED (2003)
4	Skin	Multibacillary (ENL)	M	48	MDT/MB (2009–10)

F, female; M, male; MDT, multidrug therapy; PRED, Prednisone.

After dissection, the skin biopsies were fixed in 10% formalin and processed according to standard procedures for embedding in parrafin. Paraffin section of 6 μm and/or 14 μm thickness were cut using a microtome and mounted on glass slides for further pathological analysis. Tissue sections were stained with haematoxylin-eosin for histopathological analysis and to assess the inflammatory activity in the lesions.

### Immunohistochemistry

After deparaffination and rehydration, the endogenous peroxidase activity was blocked with 0.3% H_2_O_2_ in methanol for 20 minutes. Epitopes were exposed by heat-induced antigen retrieval, in either 10mM sodium citrate buffer (pH 6.0) or 10mM Tris 1mM EDTA buffer (pH 9.0) depending on the primary antibody used (see **Table 4**). Aspecific binding of antibodies was blocked using 10% Normal Goat Serum (DAKO, Heverlee, Belgium) in phosphate buffer saline (PBS) for 30 minutes at room temperature. Primary antibodies were diluted in Normal Antibody Diluent (Immunologic, Duiven, The Netherlands) and incubated for 1 hour at room temperature. Detection was performed by incubating the sections in the secondary poly-HRP-goat anti Mouse/Rabbit/Rat IgG (Brightvision Immunologic, Duiven, The Netherlands) antibody cocktail diluted 1:1 in PBS for 30 minutes at room temperature following by incubation in 3,3- diaminobenzidine tetrahydrochloride (DAB; Vector Laboratories, Burlingame, CA) as chromogen. Counterstaining to visualize nuclei was performed by immersion in Hematoxylin for 5 minutes at room temperature, followed by differentiation in running water for 4 minutes at room temperature. Sections stained with secondary antibody alone were included as negative controls with each test. After dehydration, slides were mounted in Pertex (Histolab, Gothenburg, Sweden).

**Table 4:** Antibody, source, dilution.

Antibody	Detects	Source	Concentration/ Dilution
Polyclonal rabbit anti-rat C9 (cross-reacts with human C9)	MAC	Made in house (B.P. Morgan)	0.013 μg/μl’
Polyclonal rabbit anti-human C3d	C3d	Dako (A0063)	0.016 μg/μl *
Monoclonal mouse anti-human phosphorylated neurofilament (clone SMI31)	Axons	Sternberger Monoclonals Inc. (SMI31R)	1:1000’
Monoclonal mouse anti-LAM	LAM	Made in house (P.K. Das)	1:200’
Mouse anti- CD3	T-cells	Life technologies MHCD0300	1:500*
Mouse anti-CD68	Macrophages	PG-M1 Dako	1:200*
Mouse anti-CD20	B-cells	DAKO M755	1:400*
Mouse anti-CD21	Receptor for C3d on B- and T cells	Abcam Ab9492	1:200*

Antigen retrieval was performed with either 10mM Tris 1mM EDTA pH 9’ or 10mM Sodium Citrate pH 6*

The quantitative analysis of the immunostainings was performed with the Image Pro Plus software version 7 (Media Cybernetics Europe, Marlow, UK). Digital images of 20x magnification of the immunostainings were captured with a light microscope (BX41TF; Olympus,Center Valley, PA) using the Cell D software (Olympus). Images covering the complete skin biopsy were quantified. The surface area stained is expressed as percentage of total area examined. The error bars indicate standard error of the mean.

### Immunofluorescence

Immunofluorescence staining was performed to compare the cellular distribution of two markers in the same tissue section. Deparaffination, antigen retrieval and blocking of aspecific binding sites were performed essentially as described above. To determine which cells were C3d or MAC positive in skin lesions of leprosy patients skin sections of 6 μm were stained with the unconjugated primary antibodies against CD3^+^ T-cells, CD20^+^ B-cells or CD68^+^ macrophages together with either C3d or MAC (see **Table 4**). The unbound primary antibodies were removed by rinsing (3 × 5 min) with PBS followed by incubating with a fluorescently labeled secondary antibody for 45 min. The primary antibodies raised in rabbit (see **Table 4**) were detected with FITC (green, 488nm)-conjugated goat anti-rabbit IgG (Sigma-Aldrich, Saint Louis, MI) and the primary antibodies raised in mouse were detected with Cy3 (red, 560nm)–conjugated goat anti-mouse IgG (Sigma-Aldrich, Saint Louis, MI). Sections were air dried and mounted in Vectashield (Vector, Burlingame, CA). To determine co localization, images were captured digitally with a fluorescence microscope (DM LB2; Leica, Wetzlar, Germany) connected to a digital camera (DFC500; Leica).

To analyze the deposition of MAC on nerves, 14 μm skin sections were stained with unconjugated polyclonal rabbit anti-rat C9 and monoclonal mouse anti-human phosphorylated neurofilament (see **Table 4**). The staining was performed in the same manner as described above. The primary C9 antibody was detected with Fluorophores FITC (green, 488nm)—conjugated goat anti-rabbit (Sigma-Aldrich, Saint Louis, MI) and the SMI31 antibody was detected with the secondary antibody Cy3 (red, 550–570 nm)–conjugated goat anti-mouse (Sigma) using a Leica TCS SP8 X Confocal Microscope (LEICA Microsystems B.V., Rijswijk, The Netherlands). Z-stacks of all the positive skin areas were made using the 40x objective /1.30 Oil analyzing the 14 μm thick skin section. The images were analyzed using Leica LCS software (Leica).

### Statistical analysis

Data analysis was performed using GraphPad Prism version 5.0 (GraphPad Software Inc, San Diego, CA, USA) statistical package. Student’s *t* test was performed for statistical analysis comparing two groups. For comparison of more than two groups One way ANOVA with Bonferroni multiple comparison *post-hoc* test was used, changes were considered statistically significant for p ≤ 0.05. For the correlation analysis Shapiro-Wilk normality test was performed before using Pearson’s correlation, to determine whether the data was normally distributed.

## Results

### MAC and C3d deposition in skin of paucibacillary and multibacillary leprosy patients

**Fig 1:**
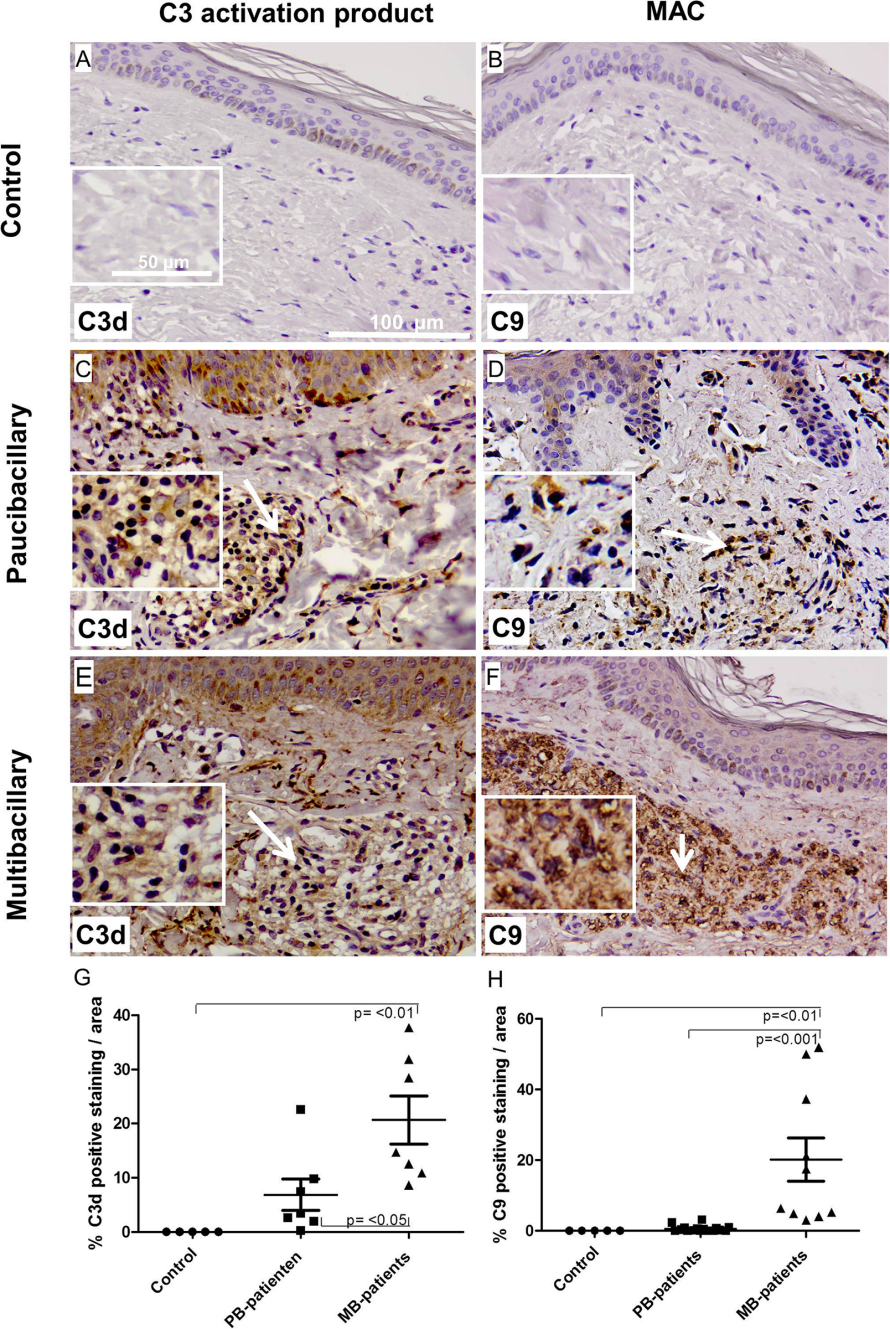
MAC and C3d deposition in skin of paucibacillary and multibacillary leprosy patients. Representative immunohistochemical stainings of skin sections from control (A and B), paucibacillary (C and D) and multibacillary (E and F) for C3d, detecting C3d, (C and E) or C9, detecting MAC (D and F). (magnification; 50 μm) showing immunoreactivity for C3d within the dermis layer of the skin of both paucibacillary (C) and multibacillary (E) (in brown) **(see arrow)**. In addition, a strong MAC immunoreactivity was found within the dermis layer of the skin of multibacillary patients (F) **(see arrow)**, indicating abundant local deposition of the active terminal complement product MAC in lesions of multibacillary patients. The control biopsies of skin and nerve are negative for C3d and C9 (A, B). Quantification of the staining (G and H), shows a significant higher amounts of C3d and MAC deposits in skin lesions of multibacillary compared to paucibacillary patients (p = <0.05 and p = <0.001 respectively). Error bars indicate standard error of the mean.

The procedure to obtain skin biopsies is less invasive than the nerve biopsies, therefore it is more commonly used in the diagnosis of leprosy patients. We carried out immunohistochemical stainings to determine whether complement is deposited in leprosy skin lesions and whether expression level is different in multibacillary compared to paucibacillary patients. Immunohistochemistry for C3d, using an anti-C3d antibody, or MAC, using an antibody against C9, which recognizes bound C9 in tissue [21], was performed on skin biopsies of controls (**Fig 1A and 1B**), paucibacillary (**Fig 1C and 1D**) and multibacillary patients (**Fig 1E and 1F**), showing immunoreactivity for C3d within the dermis of both paucibacillary (**Fig 1C, arrow**) and multibacillary (**Fig 1E, arrow**) skin. In the skin lesions of paucibacillary patients, mainly macrophages and lymphocytes were found on or in the vicinity of the positive staining, while in the skin lesions of multibacillary patients macrophages were predominantly present. In addition, extensive C9 immunoreactivity was found within the dermis of multibacillary patients’ lesions (**Fig 1F**), indicating abundant local deposition of the active terminal complement product MAC in lesions of multibacillary patients. Quantification of the staining on skin biopsies showed a significantly higher amount of C3d and MAC deposition in multibacillary compared to paucibacillary patients (p = <0.05; p = <0.001, respectively) (**Fig 1G and 1H**). Control skin biopsies were negative for C3d and MAC (**Fig 1A and 1B**).

### C3d fragments co localize with T-cells in skin lesions of paucibacillary patients

**Fig 2:**
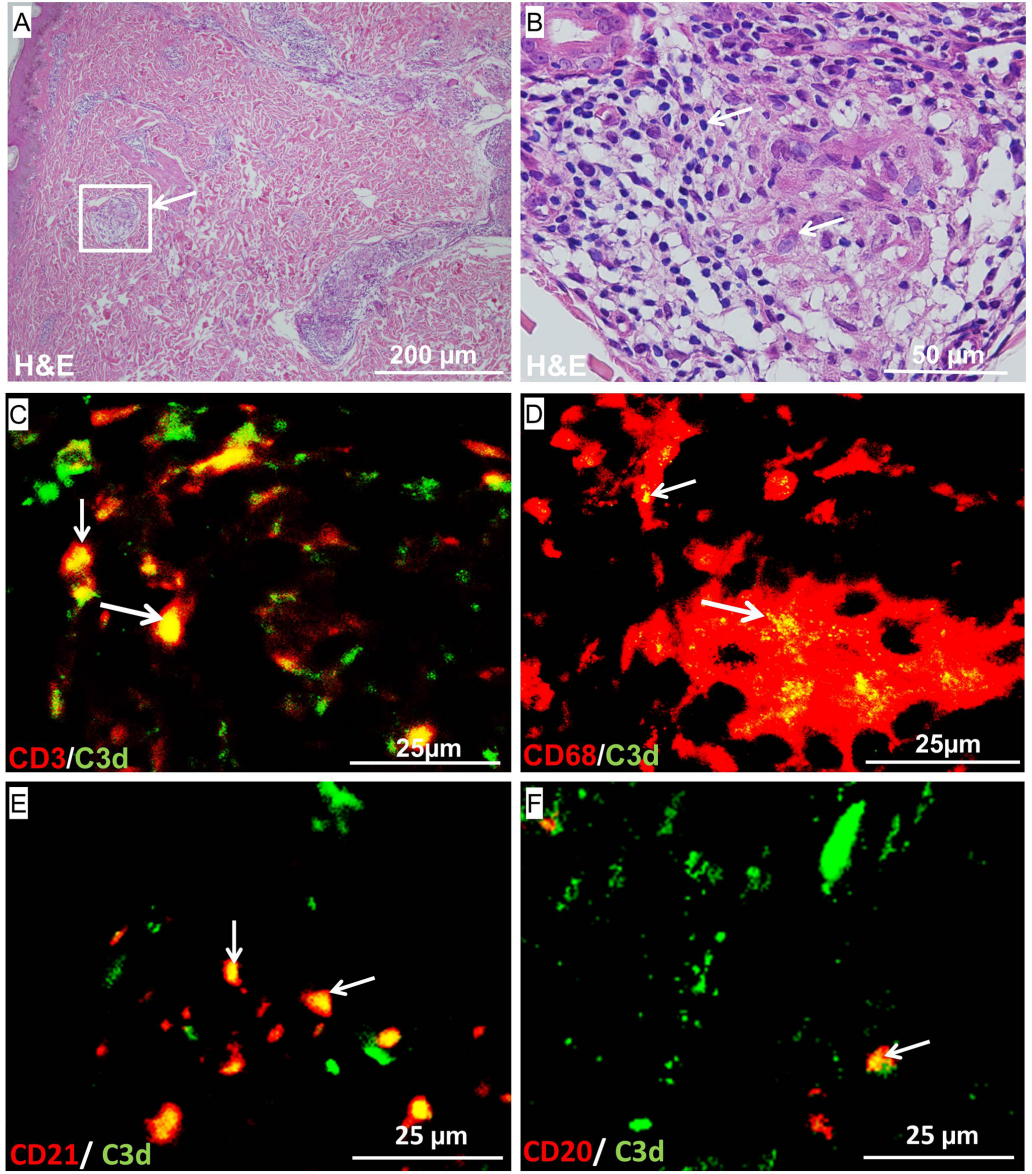
Representative stainings of skin sections from paucibacillary patients for H&E (A, B Zoom) and immunofluorescence for CD3^+^ T cells and C3d (C) CD68^+^ macrophages and C3d (D) CD21 detecting the CR2 receptor and C3d (E) or CD20^+^ B cells and C3d (F). The H&E staining shows abnormal granulomatous lesions in the skin (A). A zoom in of the H&E staining shows granulomas with epithelioid cells surrounded by lymphocytes (B) Immunofluorescence on the sections indicated that the T cells, the CR2 receptor and B-cells all co-localized with C3d in skin lesions of paucibacullary patients.

We found C3d deposited in granulomatous lesions in the skin of paucibacillary patients (**Fig 1C**). We tested whether the abundant lymphocytes and macrophages that we observed in the H&E staining in granulomatous lesions of paucibacillary patients (**Fig 2A and 2B**) were C3d positive by immunofluorescence staining. We observed that both CD3^+^ T-cells and CD68^+^ macrophages co-localized with the C3d fragment of complement in the skin lesions of paucibacillary patients (**Fig 2C and 2D**). CD68^+^ cells were occasionally MAC positive in lesions of paucibacillary patients (data not shown), but CD3^+^ T-cells were not. B- and T-cells are known to express the CR2 receptor for C3d. To confirm our findings we analyzed whether C3d also co-localized with the CR2/CD21 receptor. We observed that also CD21 co-localized with C3d in skin lesions of paucibacillary patients (**Fig 2E**).

B cells were also found to co-localize with C3d in the skin lesions of these patients, but these were not as frequently found as the C3d positive T-cells (**Fig 2F**). These findings might suggest a role for C3d in T- and B cell co-stimulation in skin lesions of paucibacillary patients.

### MAC deposited on nerves in skin lesions of multibacillary patients

**Fig 3:**
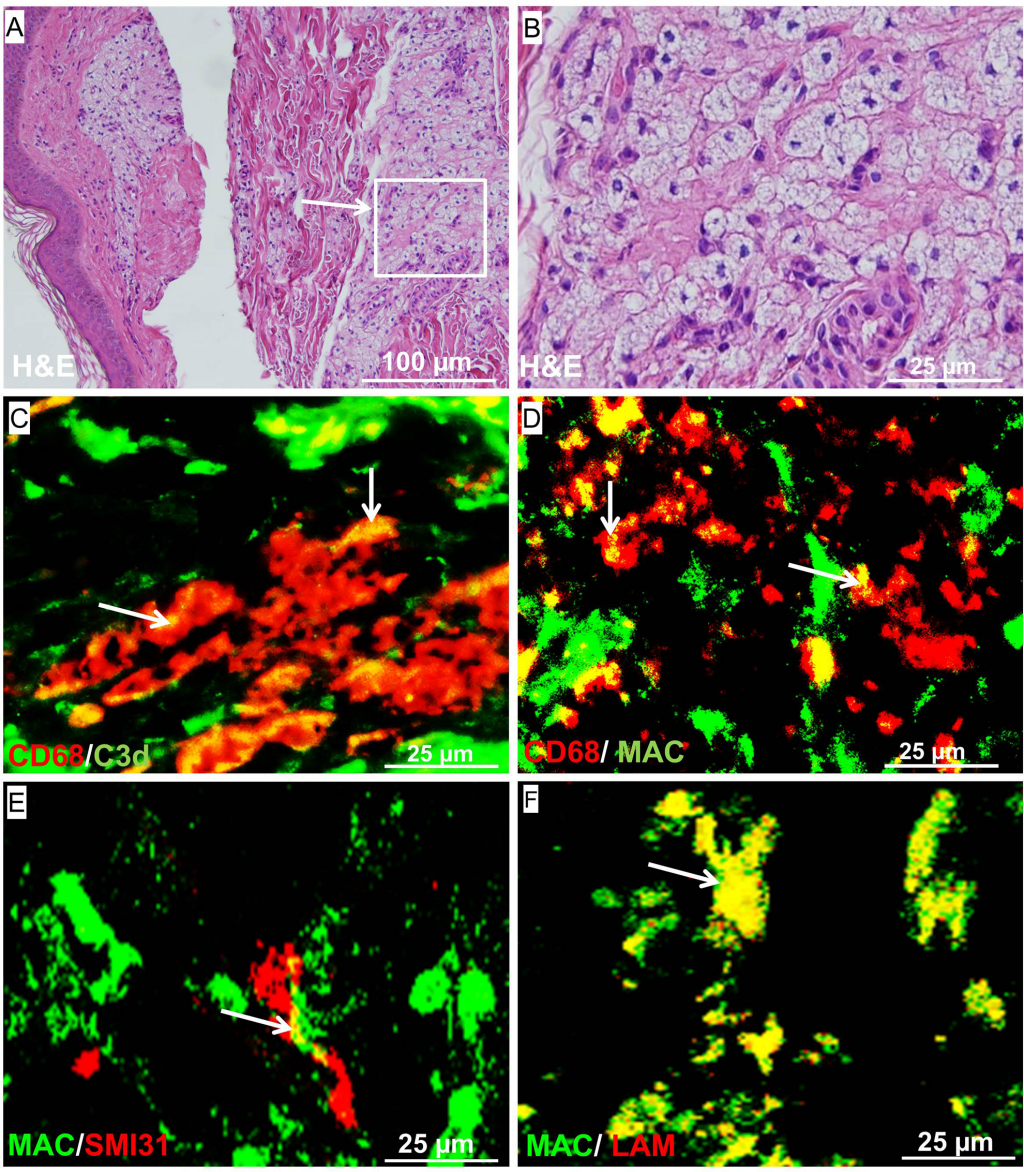
H&E staining of LL leprosy skin (magnification; 100 μm) (A) and zoom-in (magnification; 25 μm) showing giant epithelioid cells in the skin lesion. Double staining of LL leprosy skin for macrophage marker CD68 (red) with C3d (green) (C) and MAC (green) (D) showed co-localization of both complement markers with macrophages (magnification; 25 μm). Staining for MAC with the marker SMI31 that visualizes the nerves or LAM showed that these markers co-localized indicating that MAC attacks the axons in the skin and that LAM could be a trigger for the complement activation in the lesions.

We have previously shown that MAC can target the axons and cause nerve damage in a model of *M*. *leprae* induced nerve damage [17]. MAC was also found deposited on axons in nerve biopsies of leprosy patients. Here we showed that MAC is abundantly present in skin biopsies of multibacillary patients, but not in paucibacillary patients **(Fig 1D and 1F)**. We were interested in the cellular localization of MAC in skin lesions of multibacillary patients and whether MAC targets the nerve endings in the skin of leprosy patients. H&E staining on skin biopsies of multibacillary patients demonstrated numerous giant epithelioid cells in the skin lesions **(Fig 3A and 3B)**. Both complement markers C3d and MAC co-localized with CD68^+^ macrophages in skin lesions of multibacillary patients **(Fig 3C and 3D)**. In addition, we found that MAC is deposited on nerves in skin lesion **(Fig 3E)**, indicating that MAC attacks the nerves. We previously determined *in vitro* that LAM is a dominant activator of complement, here we show that also in the skin lesions MAC co-localized with *M*. *leprae* antigen LAM, suggesting that LAM triggers complement activation in these lesions **(Fig 3F)**.

### MAC deposition in skin of leprosy patients with reactions

**Fig 4:**
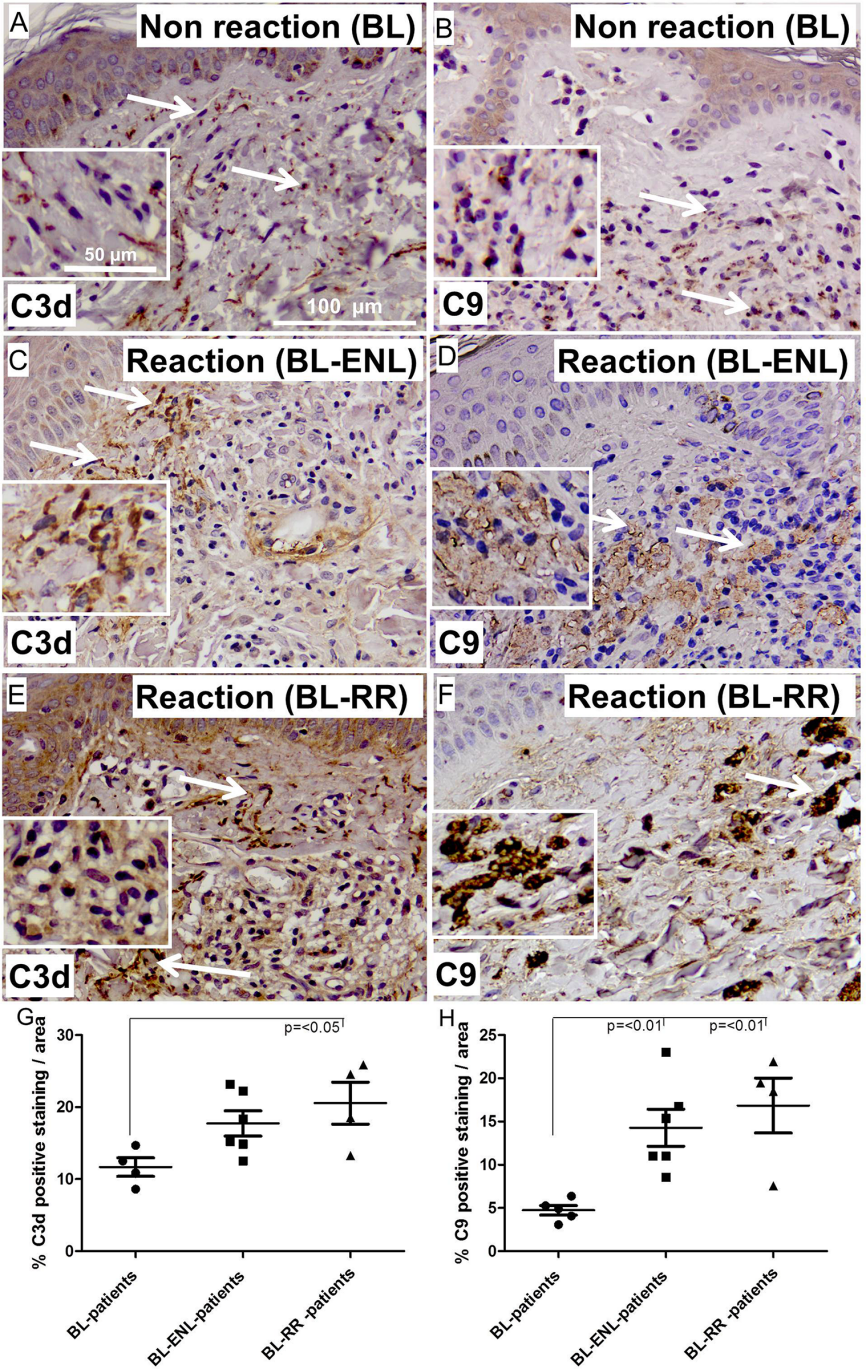
Representative immunohistochemical stainings of skin sections from BL (A and B), ENL (C and D) and RR (E and F) for C3d (A, C and E) and MAC (B, D and F) (magnification; 100 μm). Quantification of the staining (G and H), shows a significant higher amounts of C3d and MAC deposits in skin lesions of BL compared to RR patients (p = <0.05 and p = <0.01 respectively). In addition, patients that developed ENL had a higher amount of MAC deposition in the skin compared to BL patients without a reaction (p = <0.01). Error bars indicate standard error of the mean.

Reversal reaction (RR) and erythema nodusum leprosum (ENL) can result in extensive nerve damage and disabilities probably due to the immunological response to *M*. *leprae* antigens. To determine the extent of MAC and C3d deposition in skin biopsies of reaction leprosy patients we performed immunohistochemistry for C3d and C9 detecting MAC. Skin biopsies of borderline lepromatous patients without (**Fig 4A and 4B**) or with ENL (**Fig 4C and 4D)** or RR (**Fig 4E and 4F**) were analyzed. The skin biopsies of borderline lepromatous patients that developed a RR showed a significantly higher amount of C3d and MAC deposition compared borderline lepromatous patients that did not develop a reaction (p = <0.05 and p = <0.01 respectively) (**Fig 4E and 4F**). In addition, we found that patients that developed ENL showed a significantly higher amount of MAC deposition compared to borderline lepromatous patients that did not develop a reaction (p = <0.01). Quantification of the stainings indicates that patients who develop ENL or RR have a higher amount of C3d and MAC deposition in skin lesions compared to patients without reaction (**Fig 4G and 4H**).

### LAM deposition in skin of paucibacillary and multibacillary leprosy patients

**Fig 5:**
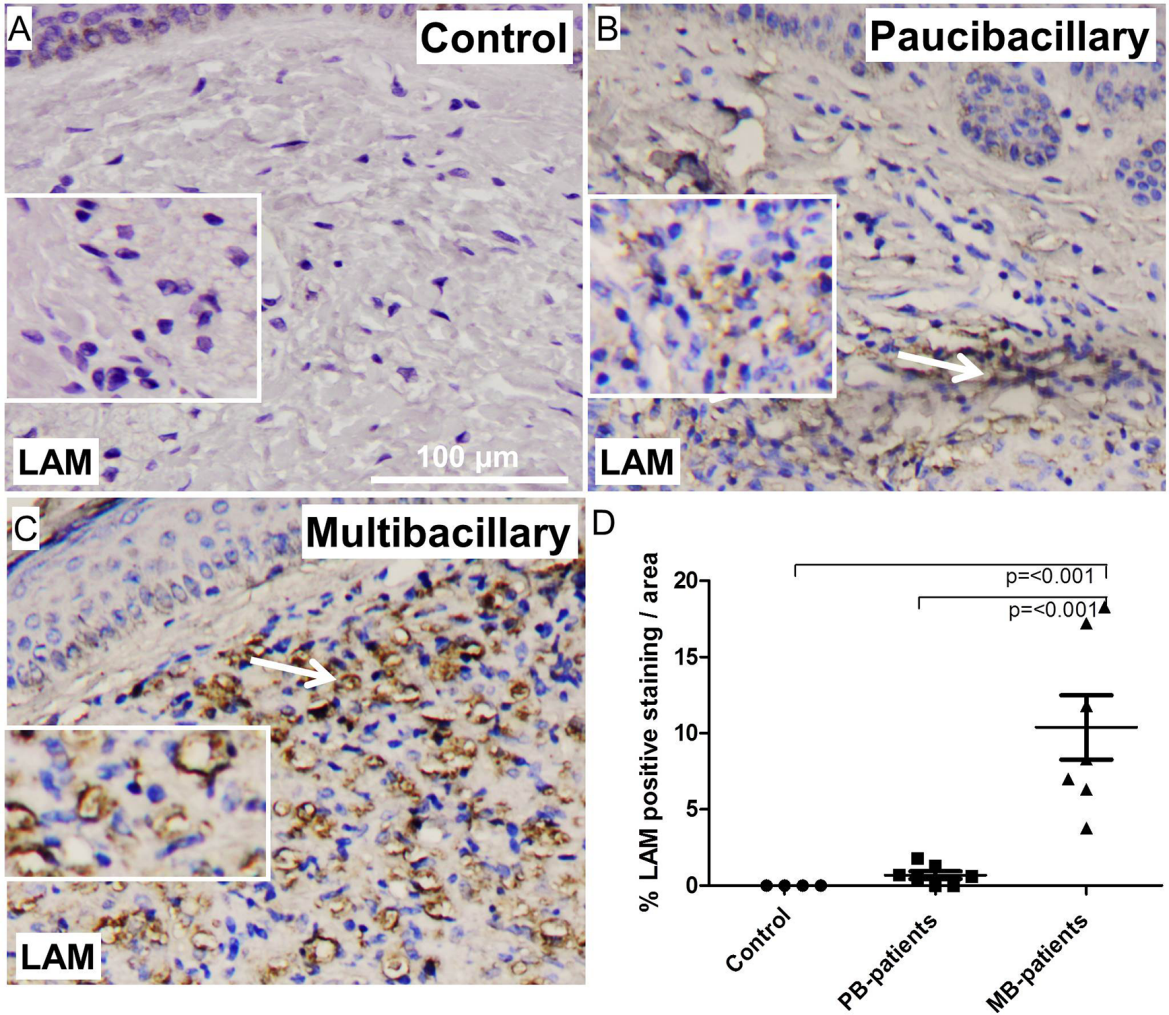
Representative immunohistochemical stainings of skin sections from Control (A), paucibacillary (B) and multibacillary (C) patients for LAM (magnification; 100 μm). Quantification of the staining (D), shows a significant higher amounts of LAM deposits in skin lesions of multibacillary compared to paucibacillary patients (p = <0.001). Error bars indicate standard error of the mean.

We previously showed that LAM is the dominant activator of complement and correlates with the amount of MAC deposition in nerve biopsies of leprosy patients. Here we determined the amount of LAM deposition in skin biopsies of paucibacillary and multibacillary patients. We found LAM deposited in both paucibacillary and multibacillary skin lesions (**Fig 5B and 5C**). Control skin were negative for LAM deposition (**Fig 5A**). Quantification of the stainings showed that skin lesions of multibacillary patients have a significantly higher amount of LAM deposition compared to paucibacillary patients (p = <0.001) (**Fig 5D**).

### LAM deposition in skin of patients with RR or ENL reaction

**Fig 6:**
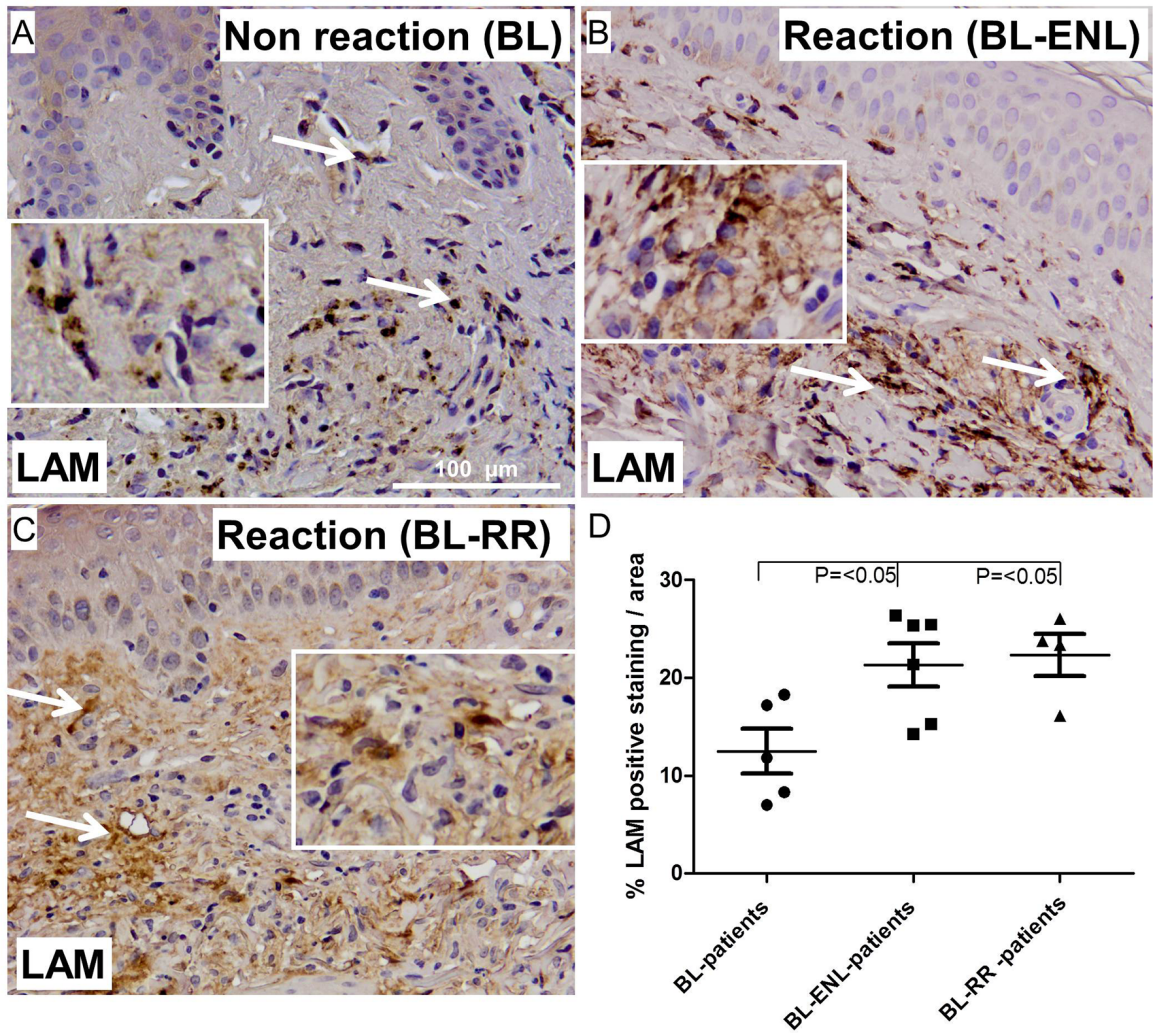
Representative immunohistochemical stainings of skin sections from BL (A), ENL (B) and RR(C) patients for LAM (magnification; 100 μm). Quantification of the staining (D), shows a significant higher amounts of LAM deposits in skin lesions of RR and ENL compared to BL patients without a reaction (p = <0.05 and p = <0.05, respectively). No statistical difference was found between ENL and RR patients in the percentage of LAM deposition in skin lesions. Error bars indicate standard error of the mean.

We also analyzed skin biopsies of reactional patients for LAM deposition. Interestingly, we found a significantly higher amount of LAM deposition in skin biopsies of borderline lepromatous patients with ENL or RR compared to borderline lepromatous patients with no reaction (p = <0.05 and p = <0.05, respectively) **(Fig 6A, 6C and 6D)**. There was no significant difference between skin biopsies of borderline lepromatous patients with ENL and borderline lepromatous patients with RR (**Fig 6A, 6B and 6D)**.

### Complement deposition is associated with the LAM and bacterial index in skin lesions

We have previously shown that there is a correlation between bacterial antigen LAM and complement deposition in nerve biopsies of leprosy patients [17]. We were interested in whether there is a link between the amount of bacterial antigens/LAM and the amount of complement activation in the skin biopsies of paucibacillary and multibacillary leprosy patients. Here, we tested whether there is a correlation between the extent of C3d staining and the bacterial index (BI) or LAM staining in corresponding skin areas.

**Fig 7:**
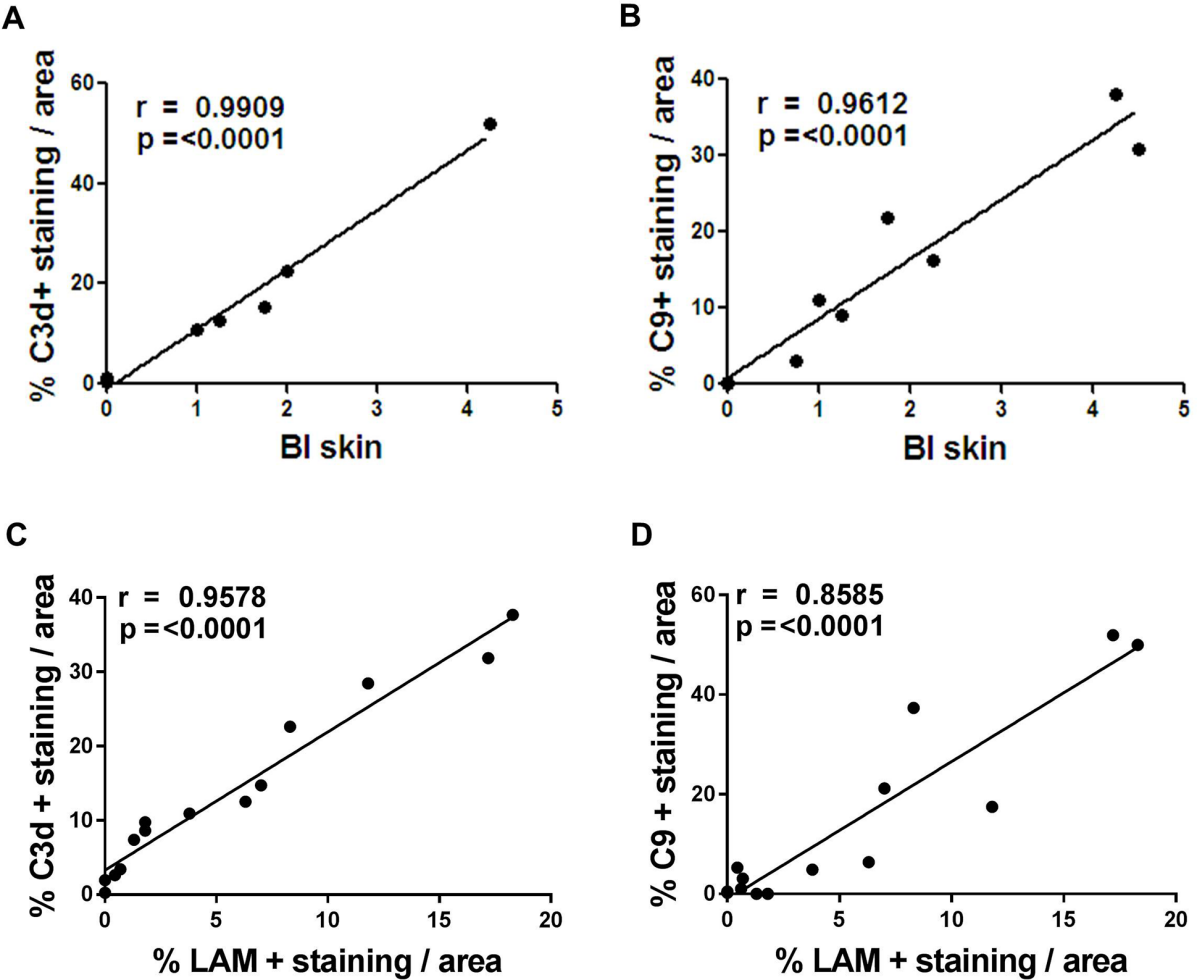
Bacterial Index (BI) and LAM deposition are associated with C3d and MAC deposition in skin lesions of leprosy patients. The amount of C3d (**a,c**) and C9 (**b,d**) immunoreactivity significantly correlated with the BI and LAM deposition in skin of paucibacillary and multibacillary (Pearson’s correlation for BI, r = 0.99909, p = <0.0001 and r = 0.9612, p = <0.0001 respectively) (Pearson’s correlation for LAM, r = 0.9578, p = <0.0001 and r = 0,8585, p = <0.0001 respectively), indicating an association between the *M*.*leprae* BI or LAM and complement activation in leprosy skin.

We found a highly significant positive correlation between the amount of C3d and BI in leprosy skin lesions (r = 0.9612, p<0.0001) **(Fig 7A)**. In line with the finding we also found that the percentage of MAC positive staining correlated with the BI in the skin lesions (r = 0.9909, p<0.0001) **(Fig 7B)**. We also found a significant association between LAM and C3d or MAC in the skin biopsies of leprosy patients (r = 0.9578, p< 0.0001 and r = 0.8585, p<0.0001 respectively) (**Fig 7D and 7E**). Overall, these data show a strong link between the presence of *M*. *leprae* antigens or more specifically LAM and complement activation products in the skin lesions of leprosy patients.

### MAC and LAM deposition in skin lesions of treated BL leprosy patients

**Fig 8:**
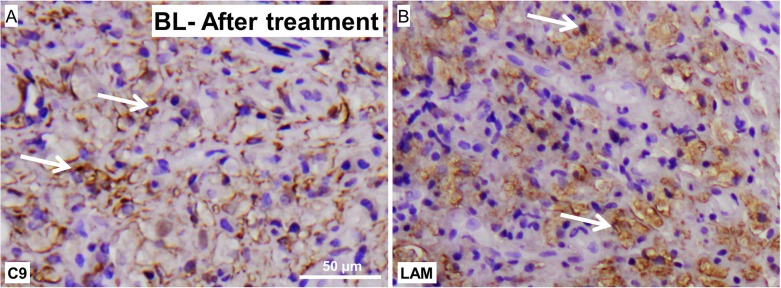
Representative staining pattern with an antibody against for LAM, detecting *M*.*leprae*, or C9, detecting MAC, in skin biopsies of a treated leprosy patients showing *M*.*leprae* antigen LAM (A) as well as MAC (B) persist in the skin after completion of treatment (magnification; 50 μm).

We also analyzed skin lesions of leprosy patients after completion of treatment. Interestingly, skin biopsies of a borderline lepromatous patients after treatment also showed high levels of MAC deposition **(Fig 8A)**. Along with these findings we found also high levels of LAM deposition in the skin of these patients **(Fig 8B)**. This data stregthens the findings that MAC is not cleared by the current treatments.

## Discussion

The aim of this study was to explore whether our previous observation in nerve biopsies can be applied on the skin lesions of leprosy patients to evaluate the association of complement activation products and persisting *M*. *leprae* antigen LAM in nerve damaging pathology in leprosy. Consequently, we first determined whether complement activation products are deposited in skin lesions of leprosy patients throughout the spectrum in relation to the presence of *M*. *leprae* antigen LAM. In addition, we examined the cellular localization of the complement activation products and whether the deposition of MAC targets the axons in the skin lesions of leprosy patients. Furthermore, we evaluated whether MAC together with *M*. *leprae* antigen LAM persists in skin lesions of patients after treatment.

We show that C3d is deposited in the center and around granulomas in skin lesions of paucibacillary patients whereas MAC deposition was rarely found in these lesions. We have to emphasize that the stainings on the skin biopsies of leprosy patients is a snap shot of the disease pathology when the patients came into the clinic. This suggests that even though MAC immunoreactivity was rarely found in paucibacillary patients, there might be an important role for MAC in nerve damage early in the disease pathology in these patients, before the adaptive immune response comes into play.

However, in paucibacillary patients C3d was found to co-localize with macrophages and T-cells in skin lesions. We suggest that C3d might play an important role in the inflammation in skin lesions of paucibacillary patients, through co-engagement of the T-cell receptor and complement receptor 2 (CR2). CR2 is normally found on the surface of B cells and is a receptor for C3d. Interestingly it has been shown by different studies that a population of T-cells also has a CR2 receptor [22–24]. C3d might bind to CR2 expressed on the surface of T-cells and, by ligand–receptor interaction result in T-cell stimulation and enhancement of the adaptive immune response. In the skin lesions of paucibacillary leprosy patients nerves were hardly detected, probably the nerves are already destroyed and the bacterial growth is controlled by the inflammation, caused by the reactive T-cells.

In skin lesions of multibacillary patients we show that complement component C3d and MAC deposition was significantly higher compared to lesions of paucibacillary patients. Also a significantly higher amount of LAM deposition was detected in the skin lesions of multibacillary patients, compared to paucibacilly patients. These findings are in line with what we previously observed in nerve biopsies of leprosy patients, where we found significantly higher amount of LAM deposition in biopsies of multibacillary compared to paucibacillary patients and LAM co-localizing with MAC on the axons [17]. In the same study we showed that MAC could be activated by *M*. *leprae* and its antigen LAM and cause nerve damage in mice, while inhibition of MAC protects the nerve. Interestingly, MAC co-localized with LAM antigen as well as nerves in the skin lesions, indicating that LAM might be a trigger for complement activation involving the axonal component. MAC immunoreactivity extended also to LAM-negative skin areas. This might suggest that the *M*. *leprae* antigen LAM activates complement in the skin and that activated complement may drift from the target site to adjacent areas attacking axons [3, 4]. It is generally assumed that the early damage in leprosy patients predominantly occurs in non-myelinated C- fibers and not in myelinated fibers. In the skin there are myelinated and non-myelinated nerves, we suggests that the first hallmark of leprosy, loss of sensory nerves in the skin, may be due to *M*. *leprae* antigen LAM which is involved in focal demyelination and complement activation. We suggest that the nerve and tissue damage is attributed to the inflammatory response generated in the surrounding tissue by LAM and MAC.

The terminal complement components are recently associated with host inflammatory responses generated by phagocytosis of complement-opsonized particles involving macrophages [25]. In skin lesions of multibacillary patients, LAM is found abundantly present in macrophages, as seen a previous study [20]. In addition, C3d and MAC were found also to co-localizing with giant macrophages in these lesions. It has been suggested that during the process of complement mediated phagocytosis MAC activates the inflammasome NLRP3 by ‘‘jumping” from the surface of complement-opsonized particles to plasma membranes of macrophages and thereby activating caspase 1 and release of IL1-b and IL-18 [20]. Irrespective of the mechanism, inflammasome activation plays an important role in the adaptive immune response including recruiting leukocytes to the site of phagocytosis. This mechanism might be involved in the skin lesions of multibacillary patients, where MAC is shown to be on macrophages and might contribute to the nerve damage.

Previous studies have shown that a number of leprosy patients experienced a reaction, RR or ENL, after the completion of 1 or 2 year of MDT. RR are severe and of longer duration and are mainly associated with neuritis [26], and occurs mainly in borderline patients (BT, mid-borderline and BL). Because acute nerve damage occurs during RR reactions accompanied by and cell-mediated immunity, a role for the immune system in causing nerve damage during RR has long been suspected [27].

Here, we show that complement activation products C3d and MAC are deposited in skin lesions of RR patients. We suggest that both C3d and MAC play an important role in the nerve damage in the skin. It is likely that C3d co-stimulates auto reactive T-cells whereas MAC lysis *M*. *leprae* infected cells to control the growth of *M*. *leprae* bacilli in the lesions. We suggest that this inflammatory environment amplifies nerve damage via the release of antigens and continuous activation of complement, resulting in MAC deposition, which targets the axons.

Leprosy patients with BL and LL forms might experience ENL. During an ENL reaction, neuritis may occur and cause permanent loss of function of the nerves. The neuritis may be less aggressive than during a RR, but is still an important problem during ENL. In ENL patients, it is suggested that inflammatory cytokines are at least partially responsible for the clinical manifestations [28, 29]. In addition, it is suggested that antigen antibody complexes are involved in the complement activation occurring in ENL and thus are involved in complement-mediated inflammation. Interestingly, skin lesions of ENL patients show an increased amount of MAC deposition compared to BL patients with no reaction, suggesting a role for complement in reaction patients. This is in line with a recent study that shows increased immunoreactivity for C1q in skin lesions of both RR and ENL patients also proving increased activation of complement in reaction patients [30]. We also found that LAM deposition was also significantly higher in skin lesions of ENL and RR patients compared to BL patients with no reaction. We previously showed that there is a correlation between the BI or LAM and MAC deposition in nerves of leprosy patients [17]. Here, we show that both the BI and LAM also correlate with C3d and MAC deposition in skin of leprosy patients indicating a strong link between the presence of *M*. *leprae* antigens in skin and complement activation. Bacterial antigen LAM can be a trigger for complement activation even after the patients complete treatment. In a previous study, LAM deposits were detected in lesions of leprosy patients after treatment [20]. We found an extensive amount of both MAC and LAM deposition in skin lesions of BL patients after treatment, indicating that treatment does not affect complement activation in the patients. This data strengthens the findings that LAM is not cleared by the current treatments. In view of our previous findings that link bacterial antigen LAM with MAC deposition and that MAC could target the axons, we suggest that complement might play an important role in *M*. *leprae* pathology if the antigens are not completely cleared from tissue after treatment. We suggest that complement mainly gets activated in tissue of multibacillary and reaction leprosy patients via the lectin pathway due to bacterial antigen LAM that triggers this pathway of the complement system by binding of MBL or ficolins that come from the circulation and results in MAC deposition on nerves in skin lesions, causing nerve damage.

In summary, complement activation products are found abundantly deposited in skin lesions of leprosy patients. Skin lesions of multibacillary and reactional leprosy patients and even after treatment were positive for MAC. We propose the following model: In multibacillary patients and reactional leprosy patients LAM is the initial trigger for complement activation and this results in MAC deposition on nerves in skin lesions, causing nerve damage and inflammation. In paucibacillary patients MAC deposition is absent to low. However, C3d positive T-cells are found in and around granulomas in skin lesions, suggesting a possible role for C3d in co-stimulation by binding CR2 on T-cells resulting in an enhanced immune response.

Our data suggests an important role for complement in *M*. *leprae* pathology in the skin lesions of leprosy patients by either causing nerve damage via MAC deposition or modulating the adaptive immune response through co-stimulation of T-cells via C3d. It should be noted that this is a retrospective study; a follow up study, including the longitudinal analysis of skin biopsies, will be important for a global and definite conclusion.
